# Novel PEI/Poly-γ-Gutamic Acid Nanoparticles for High Efficient siRNA and Plasmid DNA Co-Delivery

**DOI:** 10.3390/molecules22010086

**Published:** 2017-01-04

**Authors:** Shu-Fen Peng, Hung-Kun Hsu, Chun-Cheng Lin, Ya-Ming Cheng, Kuang-Hsing Hsu

**Affiliations:** 1 Department of Biological Science and Technology, China Medical University, Taichung 40402, Taiwan; s5439003@hotmail.com; 2 Department of Medical Research, China Medical University Hospital, Taichung 40402, Taiwan; 3 Institute of Bioinformatics and Structural Biology, National Tsing-Hua University, Hsinchu 30013, Taiwan; d947624@gmail.com; 4 Department of Chemistry, National Tsing Hua University, Hsinchu 30013, Taiwan; cclin66@mx.nthu.edu.tw; 5 Department of Agronomy, National Chung Hsing University, Taichung 40402, Taiwan; ymcheng@dragon.nchu.edu.tw

**Keywords:** polyethylenimine, poly-γ-glutamic acid, gene delivery, siRNA delivery, dual delivery nanoparticle

## Abstract

The efficient delivery of sufficient amounts of nucleic acids into target cells is critical for successful gene therapy and gene knockdown. The DNA/siRNA co-delivery system has been considered a promising approach for cancer therapy to simultaneously express and inhibit tumor suppressor genes and overexpressed oncogenes, respectively, triggering synergistic anti-cancer effects. Polyethylenimine (PEI) has been identified as an efficient non-viral vector for transgene expression. In this study, we created a very high efficient DNA/siRNA co-delivery system by incorporating a negatively-charged poly-γ-glutamic acid (γ-PGA) into PEI/nucleic acid complexes. Spherical nanoparticles with about 200 nm diameter were formed by mixing PEI/plasmid DNA/siRNA/γ-PGA (dual delivery nanoparticles; DDNPs) with specific ratio (N/P/C ratio) and the particles present positive surface charge under all manufacturing conditions. The gel retardation assay shows both nucleic acids were effectively condensed by PEI, even at low N/P ratios. The PEI-based DDNPs reveal excellent DNA/siRNA transfection efficiency in the human hepatoma cell line (Hep 3B) by simultaneously providing high transgene expression efficiency and high siRNA silencing effect. The results indicated that DDNP can be an effective tool for gene therapy against hepatoma.

## 1. Introduction

Gene therapy is a promising strategy to treat challenging diseases by correcting or silencing defective genes. The therapeutic effects come from introducing genetic materials (DNA or RNA) to encode the correct version of mRNA and proteins that is missed/mutated in abnormal cells [1,2,3]. In addition to transfect cells with plasmid DNA carrying a specific gene, introducing the small interference RNA (siRNA) is an alternative gene therapy which can knockdown the overexpressed defective genes [4,5,6]. The mechanism of the RNA interference (RNAi) technique is to induce gene-specific silencing via the cleavage of mRNA by RNA complexed with Dicer and other nucleases [7,8].

A pivotal challenge of gene-based therapy is to development safe and effective delivery vehicles. Gene delivery vehicles are categorized into viral and non-viral vectors [9,10]. Although viral vectors are efficient in gene delivery, there are also several drawbacks, e.g., limited DNA packaging capacity, difficulty of vector production, and the safety issues such as virus insertion caused mutagenesis, uncontrolled immunogenic and inflammatory responses. These drawbacks have seriously limited its application [11,12]. The common non-viral vectors are made by using the cationic liposomes or cationic polymers to encapsulate nucleic acid (DNA or RNA molecules) to form nanocomplexes. They can spontaneously condense DNA, protect DNA from DNase degradation, and the resulting nanocomplexes can enter cells via endocytosis/phagocytosis pathways or direct fusion/penetration of cytoplasmic membrane. Non-viral vectors exhibit low immunogenicity and have the potential of overcoming many limitations of viral vectors, particularly the safety problem [13]. However, how to improve the low transfection efficiency associated with non-viral vectors (polymer/DNA) has been the fundamental challenge in gene therapy.

Since 1995, polyethylenimine (PEI) is the most widely used cationic polymer for nucleic acid delivery in vitro and in vivo [9,14,15,16,17,18]. Its molecular weight or structure (linear or branched) can influence the transfection efficiency [19]. The advantages of PEI include its ability to condense and protect DNA and provides the proton buffering capacity for endosomal escaping [15,20]. Furthermore, the application of PEIs has also been extended towards siRNA delivery recently [21,22]. However, the high cytotoxicity of PEI becomes a major concern when utilizing high molecular weight or high N/P ratio of PEI to deliver DNA or siRNA into mammalian cells [23,24]. To overcome this drawback, several approaches are made such as using the lower molecular weight PEI, conjugating with biocompatible moieties (e.g., PEG), or a biocompatible or biodegradable polymers [25,26,27].

Poly-γ-glutamic acid, a naturally occurring polypeptide, is a biodegradable and non-toxic polymer [28]. In our previous studies, γ-PGA has been introduced into chitosan (CS)/DNA complexes to be used for gene delivery. By incorporating the negatively charged γ-PGA in CS/DNA complexes, the cellular uptake and the transfection efficiency can be significantly enhanced [29,30,31]. CS/γ-PGA complexes are also excellent siRNA transfection vehicles in which the anionic γ-PGA will assist the intracellular release of siRNA, resulting in an enhancement in gene silent effects [32]. Advanced studies demonstrated that the free N-terminal glutamyl group of γ-PGA on the surface of CS/DNA (siRNA)/γ-PGA is recognized by glutamyl transpeptidase on the cell membrane to result in significantly increased cellular uptake and transgene expression (or gene silencing) [31]. The enhanced transfection efficiency of γ-PGA also has been shown in the ternary complexes composed of PEI, DNA, and γ-PGA, whose surface was coated with negatively charged γ-PGA [26].

Multiple therapeutic agents simultaneously delivered within one delivery system may provide a chance to develop an effective combinational therapy. Dual nucleic acid (DNA and siRNA) co-delivery system can simultaneously target and manipulate multiple intracellular components to drastically change the cellular behavior or disease progression [33,34]. An arginine-rich oligopeptide-grafted branched PEI modified with polyethylene glycol (P(SiDAAr)5P3) has been used as gene carriers for breast cancer therapy and had been demonstrated that the polyplexes exhibit significantly lower material induced toxicity and high gene transfection efficiency [35]. Gold nanoparticles coated with degradable polymer by layer-by-layer method have been used to co-deliver DNA and short interfering RNA (siRNA) for human brain cancer therapy [36]. Copolymer composed of poly (spermine ketal ester) (PSKE) and poly (spermine ester) (PSE) at equal ratio was used to deliver both DNA and siRNA and was attributed to optimal gene expression and gene silencing [37].

In this study, we present a novel effective PEI-based delivery system for co-delivery of gene and siRNA within one nanovehicle (dual-delivery nanoparticle, DDNP), which in the future may become a useful tool for anti-cancer gene therapy, providing synergistic therapeutic effects via simultaneous expression and silencing of target genes. Here we examine the expression patterns of GFP and DsRed fluorescent proteins as an experimental model to evaluate our dual-delivery nanoparticle (DDNP). The characteristics of the test nanoparticles were examined by dynamic light scattering (DLS) and transmission electron microscopy (TEM). The internalization efficiency was examined using a confocal laser scanning microscope (CLSM) and flow cytometry. The exact gene expression and siRNA knockdown efficiency was evaluated by fluorescence microscopy and flow cytometry.

## 2. Results

In this study, we utilize the high-transfection efficiency PEI and polyanionic γ-PGA to complex with DNA/siRNA to develop DDNPs or γ-DDNPs for hepatoma cancer cell therapy. In order to monitor the effects of transgene expression or gene knockdown, pDsRed plasmid and GFP-siRNA were used, respectively, in the nanoparticle preparation. The characteristics of DDNPs and γ-DDNPs were investigated an in vitro experiments were determined in this study.

### 2.1. Characterization, Cellular Uptake, and Transfection Efficiency of DDNPs

**Figure 1:**
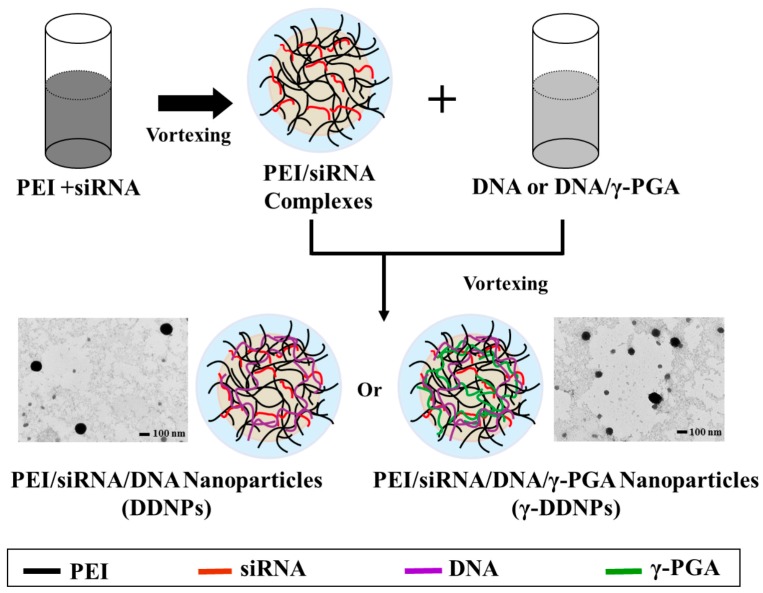
Schematic illustration of the preparation processes of DDNPs and γ-DDNPs. An aqueous siRNA solution was complexed with different amount of PEI to be followed by mixing with plasmid DNA or plasmid/γ-PGA by vortexing to form DDNPs or γ-DDNPs, respectively. The TEM images of DDNPs or γ-DDNPs were shown.

Combining the advantages of branched PEI with poly-γ-glutamic acid, a dual nucleic acid co-delivery system (DDNPs or γ-DDNPs) encapsulating DNA and siRNA was developed in this study. The DDNPs was prepared first by mixing the PEI with siRNA, and then the resulting complexes were vortexed again with DNA- or DNA/γ-PGA-solution to form a dual nucleic acid co-delivery nanoparticle (DDNPs or γ-DDNPs) as shown in Figure 1.

The characteristics of DDNPs were evaluated by different methods. The sizes of the prepared DDNPs at N/P ratio ranging from 3/1 to 10/1 were about 180–200 nm in diameter, with wide size distributions (i.e., a high polydispersity index; PDI). The zeta potential of the test nanoparticles at N/P ratio of 3/1–10/1 showed that the net charge on the particle surface was positive. With increasing N/P ratio (from 3/1 to 10/1), the zeta potentials (39.9 to 47.8 mV) increased in parallel as a result of presence of more cationic group-carrying PEI being incorporated into the nanoparticles (Table 1).

**Figure 2:**
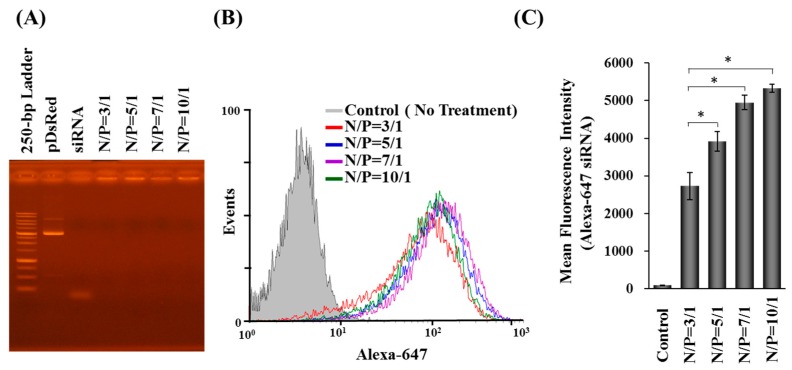
The binding capacity and cellular uptake of DDNPs. The nanoparticles were prepared by using different amount of PEI, plasmid and siRNA. The binding capacity between PEI and nucleic acid were analyzed by electrophoresis (**A**); The cellular uptake diagram (**B**) and fluorescent intensity (**C**) of nanoparticles composed of Alexa Fluor-647 labeled siRNA were examined by flow cytometry. * *p* < 0.05, significant difference between the group with various N/P ratio and the group with N/P ratio of 3/1 as analyzed by Student’s t test.

The binding capacity between branched PEI and nucleic acids (DNA and siRNA) was determined by a gel retardation assay. As shown in Figure 2A, the mobility of plasmid DNA and siRNA in all test nanoparticles was retarded irrespective of N/P ratio, indicating that the plasmid and siRNA can be stably complexed with the branched PEI.

In order to quantify the amount of internalized nanoparticles, Alexa Fluor-647-labeled siRNA was used to prepare nanoparticles to transfect Hep 3B cells and then analyzed by flow cytometry. The mean fluorescence intensities (MFI) of cells with internalized Alexa Fluor-647-labeled nanoparticles were enhanced, in parallel with increases in N/P ratio of nanoparticles, indicating that nanoparticles with a higher N/P ratio can deliver more siRNA into cells (Figure 2B,C). About 90% of cells uptook the nanoparticles (Figure 2B).

**Figure 3:**
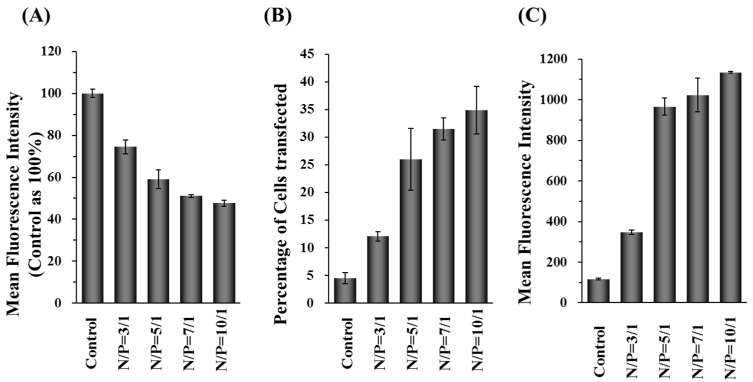
The siRNA silencing effects and transgene (DsRed) expressions of Hep 3B cells after transfected with DDNPs. The GFP fluorescent intensity were knocked down by GFP-siRNA delivered by DDNPs prepared with various N/P ratios (**A**); The percentage of DsRed gene transfected cells (**B**) and the amount of DsRed expression (**C**) was evaluated after cells were transfected with DDNPs prepared with different N/P ratios. The control group denotes the group of cells without any treatment.

Either the green or red fluorescent protein (GFP or DsRed) was used as indicators to evaluate the efficiency of the dual-delivery nanoparticles. The decreasing level of GFP or the increasing level of DsRed signal were monitored by flow cytometry after incubating the GFP-siRNA or DsRed gene containing nanovehicle with Hep 3B cells. As shown in Figure 3A, the fluorescent intensities of GFP in GFP-expressing Hep 3B cells were inhibited by 20%–50% after the incubation with GFP-siRNA carrying DDNPs and variable amount of PEI (N/P ratio = 3/1 to 10/1). The DsRed gene transfection efficiency was assessed by the percentage and fluorescence intensity of cells transfected with DDNPs. The Hep 3B cells transfected with DDNP at N/P ratio of 3/1 showed low cellular transfection efficiency (~12%) with low level of DsRed expression (Figure 3B,C). Comparing the results of DDNPs at N/P ratio = 3/1, the nanoparticles at N/P ratios from 5/1 to 10/1 enhanced the DsRed gene transfection efficiency 2- to 3-fold (25%~35%) (Figure 3B). The mean fluorescent intensities (MFI) of expressed DsRed protein increased drastically in the groups treated with DDNPs at N/P ratios of 5/1, 7/1, or 10/1 in Hep 3B cells (Figure 3C). These results indicated that our DDNPs can exert high siRNA silencing and moderate foreign gene transfection performance simultaneously.

**Figure 4:**
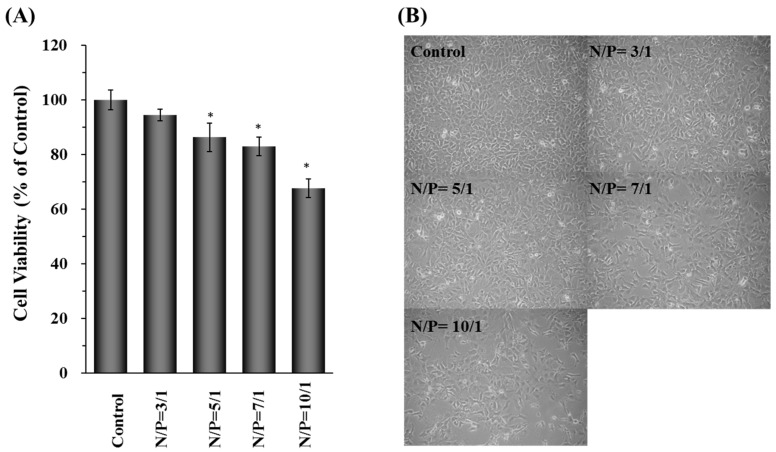
Cell viabilities (**A**) and cell morphological changes (**B**) of Hep 3B cells after incubated with DDNPs which were prepared with various N/P ratios. The control group denotes the group of cells without any treatment. * *p* < 0.05, significant difference between the group with various N/P ratio and the control group as analyzed by Student’s t test.

The above results showed that the gene silencing and transfection efficiency of DDNPs were dependent on the N/P ratios of nanoparticles. The cells transfected nanoparticles with higher N/P ratio resulted in better gene silencing and gene transfection efficiency outcomes. However, along with excellent efficiency comes higher cytotoxicity at high N/P ratios due to the presence of more cationic functional groups encapsulated inside the nanoparticles (Figure 4A).

The cells transfected with N/P ratio of 3/1, 5/1 or 7/1 exhibited acceptable cell viabilities in Hep 3B cells, showing little influence on cell number and morphological changes after DDNP treatment. When the N/P ratio reached 10/1, the cell viability decreased to 70%. Under the microscope, a trend of decreasing cell number and changing normal cell morphology was observed following the incubation with nanoparticles the increased N/P ratio (from 7/1 to 10/1), indicating that redundant PEI already interferes with the proliferation and viability of Hep 3B cells (Figure 4B). Therefore, the nanoparticles with N/P ratio equal to 5/1 were chosen to avoid cytotoxicity problems.

### 2.2. Characterization, Cellular Uptake, and Transfection Efficiency of Dual-Deliver Nanoparticles Incorporating γ-PGA (γ-DDNPs)

In order to enhance the gene expression efficiency, polyanionic γ-PGA was incorporated into the DDNPs with N/P ratio of 5/1. After incorporating γ-PGA, the γ-DDNPs with N/P/C ratios of 5/1/1 and 5/1/2 exhibited 165.4 ± 2.3 and 180.3 ± 8.2 nm, respectively, (Table 2) and a narrow size distribution (PDI). The γ-DDNPs also presented positive charges (36–40 mV) on their surface.

The morphology of γ-DDNPs with N/P/C ratios of 5/1/0 and 5/1/1 was examined by TEM (Figure 1). Both the DDNPs and γ-DDNPs were spherical in shape. The TEM observations of the nanoparticles also confirmed the size distribution measured by Zetasizer.

**Figure 5:**
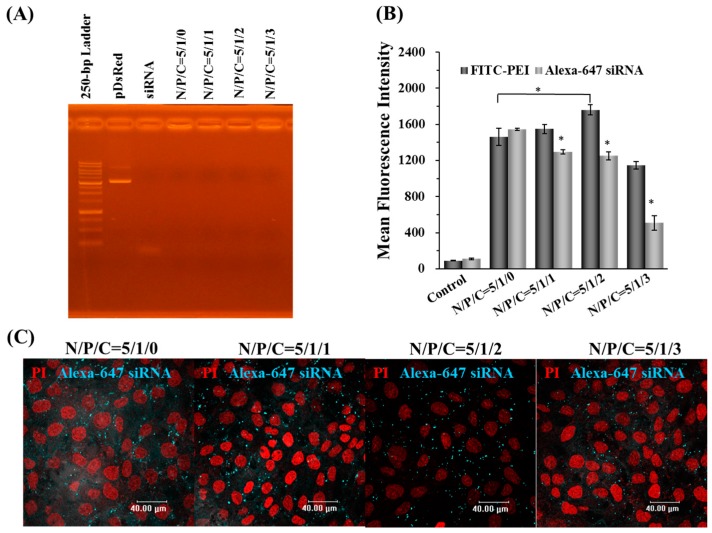
The stability and cellular uptake of PEI/dual nucleic acids/γ-PGA nanoparticles (γ-DDNPs). The nanoparticles were prepared by using different amount of PEI, plasmid DNA, siRNA, and γ-PGA. The stability of different N/P/C ration were analyzed under electrophoresis (**A**). Total cellular uptake of γ-DDNPs (FITC- or Alexa Fluor-647 labeled) with varying N/P/C ratio from 5/1/0 to 5/1/3 were analyzed by flow cytometry (**B**). After incubated with γ-DDNPs of different N/P/C ratios associated with varying N/P/C ratio (5/1/0 to 5/1/3), the fluorescence images of cells were photographed with fluorescence microscope (**C**). The control group denotes the group of cells without any treatment. * *p* < 0.05, significant difference between the test groups and the group with N/P/C ratio of 5/1/0 as analyzed by Student’s t test.

The gel retardation results indicated that the anionic γ-PGA did not affect the nucleic acid binding capacity of PEI, as the results showed no free DNA or siRNA molecules were associated with nanoparticles under the electric field with N/P/C ratios from 5/1/0 to 5/1/3. This results indicate γ-DDNPs can form stable conformations on the nanometer scale (Figure 5A).

The cellular uptake of γ-DDNPs were investigated by flow cytometry and CLSM. After 2-h post-transfection, the fluorescence intensity of the internalized FITC- and Alexa Fluor-647-labeled nanoparticles were quantified using flow cytometry. As shown in Figure 5B, the FITC-PEI fluorescence signal indicated that the total amount of γ-DDNPs internalized into cells were significantly enhanced at the N/P/C ratio of 5/1/2 compared to that of 5/1/0 (*p* < 0.05). The fluorescence intensity of the internalized Alexa Fluor-647 siRNA signal was significantly decreased with an increase in γ-PGA ratio (*p* < 0.05) (Figure 5B).

CLSM was used to visualize the cellular uptake of Alexa Fluor-647-labeled γ-DDNPs. The cell fluorescence images after incubation with nanoparticles of different N/P/C ratios were shown in Figure 5C. After 2 h post-transfection, the Alexa Fluor-647 fluorescence intensity decreased when the γ-PGA ratio increased.

**Figure 6:**
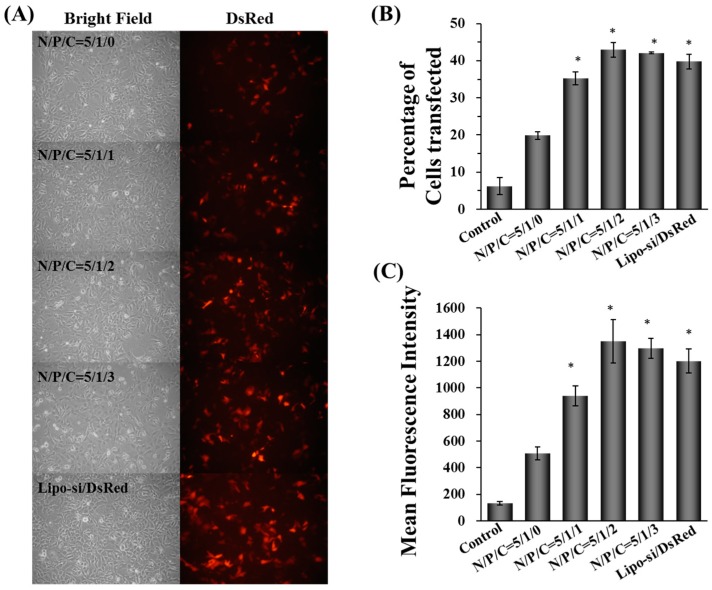
The fluorescent image of DsRed protein expressing Hep 3B cells after 48 h transfection with γ-DDNPs containing various ratio of γ-PGA (**A**). The transfection efficiency of DsRed gene (**B**) and mean fluorescent intensity (**C**) of Hep 3B cells transfected with γ-DDNPs containing various N/P/C ratios for 48 h were examined by flow cytometry. The control group denotes the group of cells without any treatment. * *p* < 0.05, significant difference between the test groups and the group with N/P ratio of 5/1/0 as analyzed by Student’s t test.

As shown in Figure 6A, the DsRed expression levels of cells transfected with γ-DDNPs with various γ-PGA ratios (γ-DDNPs) were significantly higher than those without γ-PGA (DDNPs). Flow cytometry was used to determine the exact transfection efficiency after 48 h of transfection with γ-DDNPs containing DsRed gene. As shown in Figure 6B, only up to 20% of Hep 3B cells expressed DsRed gene when transfected with the nanoparticles without γ-PGA (DDNP) (N/P/C ratio of 5/1/0). Incorporating γ-PGA into nanoparticles (γ-DDNPs, N/P/C ratios from 5/1/1 to 5/1/3), increased the transfection efficiency (35%–45%) of DsRed (*p* < 0.05). This result was confirmed by flow cytometry where the DsRed fluorescent intensity of Hep 3B cells transfected with nanoparticles containing γ-PGA (γ-DDNPs) showed significantly higher fluorescent intensity than those transfected with the nanoparticles without γ-PGA (DDNP) (*p* < 0.05) (Figure 6C).

**Figure 7:**
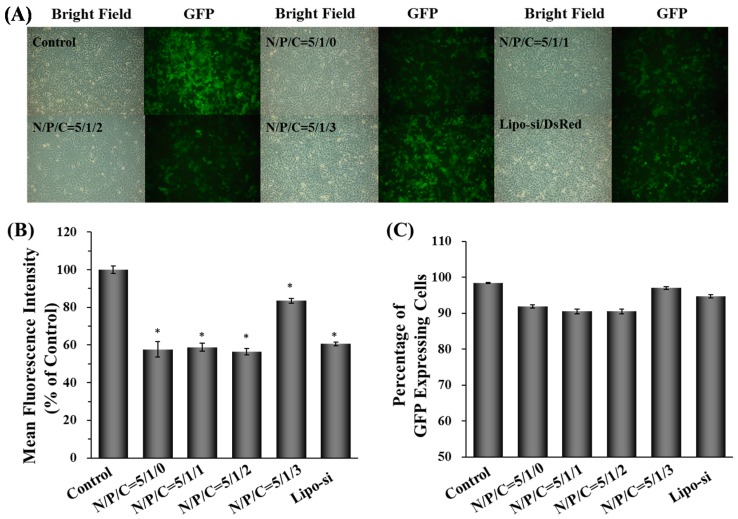
The gene silencing ability of γ-DDNPs. The GFP expressing-Hep 3B cells transfected with γ-DDNPs with different N/P/C ratios for 48 h. The fluorescent cellular images were taken after 48 h transfection with γ-DDNPs at different N/P/C ratios (**A**). The fluorescence intensity (**B**) and the percentage of fluorescence-expressing cells (**C**) after 48 h transfection with γ-DDNPs at different N/P/C ratios were examined by flow cytometry. The control group denotes the group of cells without any treatment. Lipo-si group means the GFP-siRNA transfection by Lipofectamine 2000. * *p* < 0.05, significant difference between the test group and the control group as analyzed by Student’s t test.

The siRNA delivery and gene knockdown ability of γ-DDNPs were also examined by the following experiments. The Hep 3B-GFP cells stably expressed GFP proteins were used as model cell to test the silencing effect of γ-DDNPs containing GFP-siRNA. The gene silencing effects were investigated by fluorescent microscope and flow cytometry. The gene silencing abilities of DDNP or γ-DDNPs are shown in Figure 7A. The GFP expression levels were obviously inhibited (>40%) after treatment with γ-DDNPs of N/P/C ratios from 5/1/0 to 5/1/2. However, a high amount of γ-PGA incorporation (N/P/C ratio of 5/1/3) resulted in poor inhibition of GFP expression. We used Lipofectamine 2000, a commercially available tranfection reagent, as positive control to compare with our γ-DDNPs. It is evident that our γ-DDNPs were outperformed by the Lipofectamine 2000 with greater GFP knockdown efficiency (Figure 7).

### 2.3. Cell Cytotoxicity of γ-DDNPs

**Figure 8:**
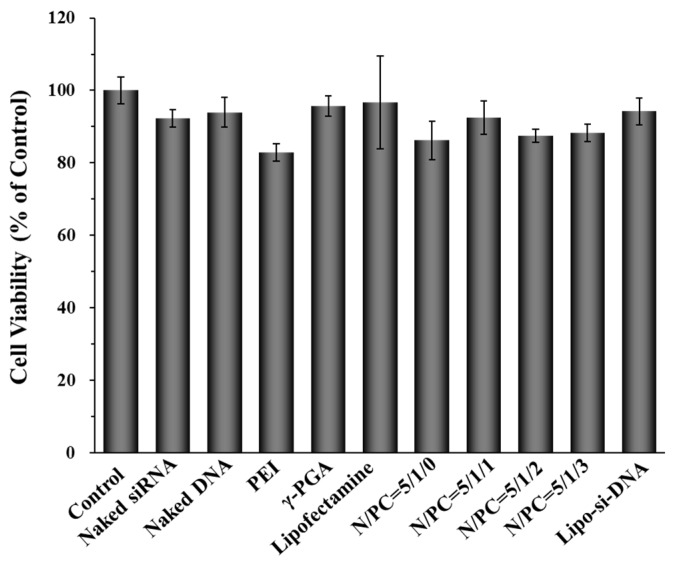
Cell cytotoxicity of raw materials and dual-delivery nanoparticles (DDNPs and γ-DDNPs) composed of different N/P/C ratios after 48 h treatment in Hep 3B cells. The control group means the group of cells without any treatment. Lipo-si-DNA group means the GFP-siRNA and pDsRed DNA transfection by Lipofectamine 2000.

The cytotoxicity of γ-DDNPs prepared with various N/P/C ratios was evaluated by MTT assays. The results are given in Figure 8. The cytotoxicity of raw materials (naked siRNA, naked plasmid DNA, γ-PGA and Lipofectamine 2000) was less than 10%. Similarly, minor cytotoxicity reduction was observed in all tested γ-DDNPs, ranging from 85% to 95%. There were only slight differences in cell viabilities with and without incorporated γ-PGA in our dual-delivery nanoparticles. These results indicated that the PEI/nucleic acid ratio of 5/1 is only slightly toxic to Hep 3B cells, which allows the γ-DDNPs to be used as a promising delivery system for hepatocellular carcinoma gene therapy.

## 3. Discussion

Gene therapy is a promising next generation medicine strategy which offers great potential to overcome diseases caused by genetic disorders or uncontrolled expression of mutant genes, and even infectious diseases caused by viruses. There are two approaches to achieve effective manipulation of gene expression, one is to transfer the entire gene sequences into cells and replace the defective version; the other way is using technologies like RNAi or antisense DNA/RNA to knock down the expression of defective or abnormally regulated genes. Both approaches need effective nucleic acid delivery vehicles to successfully transfer giant DNA/RNA molecules which accompany with strong negative charges to across the native barriers like plasma membranes and to escape from endocytic organelles.

Non-viral gene delivery vectors are potential gene vehicles such as polymeric nanoparticles and liposomes [30,38,39,40]. Polymeric nanoparticles usually consist of cationic polymers and anionic DNA/RNA sequences, where electrostatic interactions will condense the cationic polymers with their anionic cargos to form stable polymeric nanocomplexes. The DNA cargo will be released due to environmental pH changes after the polymeric nanoparticle enters into the endocytic pathways.

Polyamines, including PEI, poly-l-lysine, spermidine, and spermine, are positively charged polymers under physiological conditions. They can complex with negatively charged nucleic acid (DNA or RNA) by electrostatic interaction for gene delivery [41,42]. PEI is one of the potential polyamine-based transfection agents and it provides excellent pH-buffering capacity. When nanovehicles composed of PEI are trapped in the endosomal organelles, PEI will absorb the protons inside the organelles and cause the accumulation of water molecules that eventually disrupt the organelles’ membrane releasing the nanovehicles and their cargos into the cytoplasm (the “proton sponge effect”) [43,44]. Due to the advantages of PEI, PEI-based nanovehicles have been developed for cancer therapy [45,46,47].

DNA or RNA can electrostatically complex with cationic polymers to form nanovehicles for intracellular delivery of genetic materials. However, many non-viral gene vectors based on cationic polymer have been reported to cause cytotoxicity due to the strong electrostatic interaction of positively charged materials with cell membrane [48,49]. The cytotoxcity of PEIs are dependent on their structure and molecular weight. In general, low molecular-weight PEI reveals less cytotoxicity than high molecular-weight PEIs. Linear PEI shows low cytotoxicity than branched PEI [50]. Therefore, polyplexes composed of PEI show high gene expression in vitro and in vivo because they strongly interact with and are quickly taken up by cell surfaces due to their strong cationic properties. Furthermore, PEI provides the ability to trigger the endosomal escape which helps the nanoparticles translocate to the cytoplasm via the pH-buffering effect. However, the cationic properties of PEI will induce cytotoxicity like other cationic polymers [43,51,52]. In this study, a high amount of PEI was used to prepare nanoparticles which showed high transfection efficiency (Figure 3B,C) but also exhibited severe cytotoxicity (Figure 4A,B). One promising approach to reduce the cytotoxicity is to incorporate or recharge anionic polymers into the cationic PEI complexes [26,29,30,53]. Therefore, in this study, γ-PGA was incorporated into γ-DDNPs and resulted in the high gene/siRNA transfection efficiency with minor cytotoxicity (Figure 6 and Figure 7).

The major limitation of gene therapy is the inability to deliver the cargo successfully to the target site before or after entering cells. PEI can condense nucleic acids and form compact nanoparticles resulting in protection of nucleic acids from nuclease cleavage. The literature reports that PEI-based nanoparticles enter cells by endocytosis. The endocytosis pathway of PEI-based nanoparticles involved in cellular uptake and transgene expression is the caveolar- and clathrin-mediated pathway in HeLa cells [54,55,56]. Once taken up into cells, PEI/nucleic acid nanoparticles revealed strong buffering capacity to escape from the endosome and resulted in the release of nucleic acids (siRNA or DNA) into the cytoplasm. Finally, the siRNA directly affects the specific gene silencing and the DNA enters the nucleus and then expresses the transgene [57,58].

Several researchers have used γ-PGA as raw materials to form drug carriers for sustained release usage, or form biodegradable fibers [59,60,61]. In our study, the addition of γ-PGA to DDNPs decreased the zeta potential in a concentration-dependent manner (Table 2). The particle sizes of γ-DDNPs were not larger than DDNP; therefore, γ-PGA might have changed the internal structure of the DDNPs [29,30]. The internal nanostructure of this system will be further analyzed in our next study. As shown in Figure 5B, the polyanionic γ-PGA has an effect on enhancing cellular uptake of γ-DDNPs which is consistent with our previous studies [29,30]. As shown in Figure 5B, the fluorescence intensity of Alexa Fluor-647-siRNA fluorescent positive cells upon internalization of nanoparticles showed a trend of decreasing signal with increasing amount of γ-PGA incorporation. These results could contribute to the anionic competition between γ-PGA and siRNA during complexing with cationic PEI, resulting in an unstable siRNA binding inside the nanoparticles when incorporating large amount of γ-PGA during nanoparticle preparation. The mechanism of how γ-PGA affects the cellular uptake is not well known. However, we previously found that a γ-PGA-incorporated or coated polymer complex was taken up via a receptor-mediated endocytosis pathway, the γ-glutamyl transpeptidase-mediated pathway [29,31]. After incorporating γ-PGA into our γ-DDNPs, an enhancement of the cellular uptake of the nanoparticles was observed (Figure 6).

The ultimate goal of our research is to create an efficient tool with dual control ability which can simultaneously up- and down-regulate two specific target genes inside one cell. In this study, our dual delivery nanoparticle with low molecular-weight PEI (25 kDa) and low N/P ratio (5/1) (DDNP) can perform as an efficient dual nucleic acid delivery vehicle that shows low cytotoxicity in comparison with other nanoparticles made from high molecular-weight PEI. The introduction of γ-PGA into the DDNP system (γ-DDNPs) with N/P/C ratio of 5/1/1 exhibited improved DsRed gene transfection efficiency and high GFP knockdown effects in a hepatocellular carcinoma cell line (Hep 3B). These results indicate that the γ-DDNPs could serve as effective nucleic acid drug delivery vehicles for the future treatment of hepatoma.

## 4. Experimental Section

### 4.1. Materials

PEI (branched, MW 25 kDa), fluorescein isothiocyanate (FITC), MTT [3-(4,5-dimethylhiazol-2-yl)-2,5-diphenyltetrazolium bromide], dimethyl sulfoxide (DMSO) were purchased from Sigma-Aldrich (St. Louis, MO, USA). Lipofectamine 2000 and Lysotracker Red DND-99 were purchased from Invitrogen (Carlsbad, CA, USA). GFP-22 siRNA (sense 5’-GCAAGCUGACCCUGAAGUUCAU-3’ and antisense-5’GAACUUCAGGGUCAGCUUGCCG-3’) and AllStarts Neg. siRNA AF 647 were obtained from Qiagen (Valencia, CA, USA). Plasmid peGFP-N2 (4.7 kb) and pDsRed-Monomer-N1 (4.7 kb) that encoded green and red fluorescent protein, respectively, was purchased from Clontech (Mountain View, CA, USA). Poly-γ-glutamic acid (γ-PGA) was purchased from Challenge Bioproducts (Taichung, Taiwan).

### 4.2. Plasmid DNA Preparation

The plasmid peGFP-N2 and pDsRed-Monmer N1 used in this study were transformed into and amplified by DH5α. The plasmid DNAs were purified by Genopure Plasmid Midi Kit (Roche, Mannheim, Germany) according to the manufacturer’s instructions. The purity and integrity of plasmids were analyzed by gel electrophoresis (0.8% agarose), while their concentration was measured by UV absorption at 260 nm.

### 4.3. Preparation of Test Nanoparticles

The charge ratio (N/P/C) of test nanoparticles was expressed as the mole ratio of the amine groups (N) on PEI to the total phosphate groups (P) on DNA and siRNA, and the carboxyl groups (C) on γ-PGA. Test nanoparticles at various N/P molar ratios (3/1, 5/1, 7/1, or 10/1) were prepared by an ionic gelation method and illustrated in Figure 1. Briefly, an aqueous siRNA (1 μg) was complexed with different amounts (5.4, 9.0, 12.6 or 18.0 μg) of PEI with a final volume of 100 μL followed by mixing with 100 μL plasmid DNA (4 μg) by vortexing for 40 s. DDNPs with a molar ratio of 5/1 were selected for incorporating γ-PGA and were prepared with two separate steps. First, the PEI/siRNA complexes were prepared by using PEI solution (9.0 μg) mixed with siRNA (1 μg) by vortexing for 30 s. Second, the PEI/siRNA solutions were added into the solution containing plasmid DNA (4 μg) and varying molar ratios (0, 1, 2, or 3) of γ-PGA (25 kDa, 2, 4 or 6 μg), then thoroughly mixed for 30–60 s by vortexer to form γ-DDNPs, and left for at least 1 h at room temperature.

### 4.4. Particle Size and Zeta Potential Measurements

The size distribution and zeta potential of test nanoparticles were measured by using a Zetasizer Nano ZS (Malvern Instruments Ltd., Worcestershire, UK).

### 4.5. Gel Retardation Assay

The test nanoparticles prepared at various N/P/C ratios were examined for their ability to bind DNA and siRNA through gel electrophoresis. The prepared nanoparticles containing 0.4 μg DNA and 0.05 μg siRNA were mixed with 6× DNA loading dye and then were analyzed on a 1.0% agarose gel. After electrophoresis, the gel was stained in ethidium bromide solution and the DNA or siRNA bands were visualized by a UV box.

### 4.6. Nanoparticle Morphology Observation

An aliquot (10 μL) of the prepared nanoparticles were dropped on a carbon-coated grid and the grid was stained with 0.5% uranyl acetate and the grid was dried by air dry overnight at room temperature. The morphology and the size of the test nanoparticles were examined under a Philips CM20 transmission electron microscope (Philips Electronic Instruments, Mahwah, NJ, USA) at 120 KV.

### 4.7. Cell Culture and In Vitro Transfection

Hep 3B (human hepatoma) cells obtained from BCRC (Hsinchu, Taiwan) and Hep 3B cells expressing GFP were cultured in DMEM medium supplemented with 2.2 g/L sodium bicarbonate, 100 units/mL penicillin and 100 μg/mL streptomycin and 10% fetal bovine serum (FBS) in humidified air containing 5% CO_2_ at 37 °C [62]. Cells were sub-cultured according to BCRC recommendations.

For transfection, cells were seeded on 6-well plates at 2 × 10^5^ cells/well and transfected at next day at 70%–80% confluency. Prior to transfection, the culture medium was removed and cells were rinsed twice with transfection medium (DMEM medium without FBS). Cells were incubated in 1.2 mL transfection medium containing various N/P/C ratios of test nanoparticles or Lipofectamine 2000 (as a positive control), which carrying 2 μg DNA and 0.5 μg siRNA/well.

After 2 h transfection, the transfection media containing nanoparticles were removed, the cells rinsed twice with transfection media and refilled with FBS-containing media until analysis at 48 h after transfection. Cells were then observed under a fluorescence microscope (Carl Zeiss Optical, Chester, VA, USA) to monitor the morphological changes and to obtain the transfection efficiency.

### 4.8. Fluorescent Nanoparticle Preparation, CLSM Visualization and Flow-Cytometry Analysis

FITC-labeled (PEI) and Alexa Fluor-647 labeled (siRNA) nanoparticles were then prepared as described in Section 2.2 to track the internalization of nanoparticles by CLSM and to quantify their cellular uptake by flow cytometry, respectively. FITC-labeled PEI (FITC-PEI) were synthesized according to the methods described in the literature.

To quantify the cellular uptake of nanoparticles, cells were plated on 6-well plates and transfected with FITC- and Alexa Fluor-647-labeled nanoparticles at a concentration of 2 μg DNA and 0.5 μg siRNA/well for 2 h. After transfection, cells were trypinized and transferred to microtubes. Subsequently, cells were washed with PBS and fixed in 4% paraformaldehyde. Finally, the cells were transfer to FACS tube and analyzed by a flow cytometer.

To track the cellular uptake of nanoparticles, cells were seeded on 12-well plates with a sterile glass coverslip at 1 × 10^5^ cells/well and incubated overnight. Subsequently, cells were rinsed twice with transfection media and transfected with FITC- and Alexa-647-labeled nanoparticles. After incubation for 2 h, test samples were aspirated. Cells were then washed twice with PBS twice before they were fixed in 4% paraformaldehyde. Finally, the fixed cells were examined under a CLSM (TCS SL, Leica, Germany).

### 4.9. Percentage and Gene Expression Level of Cells Transfected

The percentage and gene expression levels of DsRed transfected cells were quantitatively assessed at 48 h after transfection by flow cytometry. Briefly, cells were treated with test nanoparticles encapsulated with pDsRed-N1. After 48 h, cells were trypsinized as described in Section 4.8. For each sample, 10,000 events were collected and fluorescence was detected. The percentage and mean fluorescence intensity of DsRed expressed cells was calculated as the events within the gate divided by the total number of events, excluding cell debris. And the DeRed expressed cells were also observed under fluorescence microscope.

### 4.10. In Vitro Gene Silencing

The gene silencing was evaluated by quantifying the silencing of GFP-expressing Hep 3B (GFP-Hep 3B) cells. For transfection, GFP-Hep 3B Cells were seeded on 6-well plates at a density of 5 × 10^5^ cells/well and transfected at next day at 60%–80% confluency. Prior to transfection, the culture medium was removed and cells were rinsed twice with transfection medium (DMEM without FBS). Cells were incubated in 1.2 mL transfection medium containing test nanoparticles or Lipofectamine 2000 (as a positive control) at 2 μg pDsRed DNA and 0.5 μg GFP siRNA /well.

The gene silencing level of GFP expressing cells were quantitatively assessed at 48 h after transfection by flow cytometry. Cells were trypsinized and then transferred to microtubes, fixed by 4% paraformaldehyde and determined the transfection efficiency by flow cytometry (BD FACSCanto flow cytometer, San Jose, CA, USA). For each sample, 10,000 events were collected and fluorescence was detected.

### 4.11. Cell Viability Assay

The cytotoxicity of test nanoparticles was evaluated in vitro using the MTT assay. Hep3B cells were seeded on 24-well plates at 3 × 10^4^ cells/well, allowed to adhere overnight and transfected by test nanoparticles containing 0.5 μg DNA and 0.125 μg siRNA. After 2 h post-transfection in transfection medium without FBS, test samples were aspirated and cells were incubated in DMEM medium with 10% FBS for another 46 h. Subsequently, cells were incubated in a DMEM medium containing 0.5 mg/mL MTT reagent for an additional 4 h. The MTT reagent was aspirated, refilled 500 μL of dimethyl sulfoxide (DMSO), and then measured in a microplate reader (Tecan Group Ltd., Männedorf, Switzerland) at a wavelength of 595 nm.

### 4.12. Statistical Analysis

Comparison between groups was analyzed by the one-tailed Student’s *t*-test (SPSS, Chicago, IL, USA). All data are presented as a mean value with its standard deviation indicated (mean ± SD). Differences were considered to be statistically significant when the *p* values were less than 0.05.

## 5. Conclusions

A PEI-based nanoparticle system incorporating γ-PGA was developed in the study as an efficient vector for dual nucleic acid delivery. The use of such a nanoparticle composed of PEI, DNA, siRNA, and γ-PGA can simultaneously enhance the expression of a delivered gene and knockdown the overexpressed gene in hepatoma Hep 3B cells. The results obtained in the study may be used for the antitumor applications.

## Figures and Tables

**Table 1:** Physicochemical properties of DDNPs prepared by different N/P ratios (*n* = 3).

N/P Ratio	Size (nm)	PDI	Zeta Potential (mV)
3/1	178.6 ± 19.8	0.44 ± 0.02	39.9 ± 5.8
5/1	197.0 ± 9.2	0.43 ± 0.03	43.6 ± 0.5
7/1	204.7 ± 3.5	0.43 ± 0.04	45.7 ± 0.6
10/1	203.4 ± 3.8	0.44 ± 0.02	47.8 ± 2.2

**Table 2:** Physicochemical properties of γ-DDNPs prepared by different N/P ratios (*n* = 3).

N/P/C Ratio	Size (nm)	PDI	Zeta Potential (mV)
5/1/0	197.0 ± 9.2	0.43 ± 0.03	43.6 ± 0.5
5/1/1	165.4 ± 2.3	0.38 ± 0.02	36.1 ± 7.3
5/1/2	180.3 ± 8.2	0.26 ± 0.03	40.0 ± 1.4
5/1/3	201.5 ± 25.9	0.15 ± 0.02	36.6 ± 1.4

## References

[1] Pack D.W., Hoffman A.S., Pun S., Stayton P.S. (2005). Design and development of polymers for gene delivery. Nat. Rev. Drug Discov..

[2] Merdan T., Kopecek J., Kissel T. (2002). Prospects for cationic polymers in gene and oligonucleotide therapy against cancer. Adv. Drug Deliv. Rev..

[3] Mulligan R.C. (1993). The basic science of gene therapy. Science.

[4] Pecot C.V., Calin G.A., Coleman R.L., Lopez-Berestein G., Sood A.K. (2011). RNA interference in the clinic: Challenges and future directions. Nat. Rev. Cancer.

[5] Van Zundert B., Brown R.H. (2017). Silencing strategies for therapy of SOD1-mediated ALS. Neurosci. Lett..

[6] Li J., Liu J., Guo N., Zhang X. (2016). Reversal of multidrug resistance in breast cancer MCF-7/ADR cells by h-R3-siMDR1-PAMAM complexes. Int. J. Pharm..

[7] Meister G., Tuschl T. (2004). Mechanisms of gene silencing by double-stranded RNA. Nature.

[8] Rana T.M. (2007). Illuminating the silence: Understanding the structure and function of small RNAs. Nat. Rev. Mol. Cell Biol..

[9] Park T.G., Jeong J.H., Kim S.W. (2006). Current status of polymeric gene delivery systems. Adv. Drug Deliv. Rev..

[10] Kataoka K., Harashima H. (2001). Gene delivery systems: Viral vs. non-viral vectors. Adv. Drug Deliv. Rev..

[11] Glover D.J., Lipps H.J., Jans D.A. (2005). Towards safe, non-viral therapeutic gene expression in humans. Nat. Rev. Genet..

[12] Woods N.B., Muessig A., Schmidt M., Flygare J., Olsson K., Salmon P., Trono D., von Kalle C., Karlsson S. (2003). Lentiviral vector transduction of NOD/SCID repopulating cells results in multiple vector integrations per transduced cell: Risk of insertional mutagenesis. Blood.

[13] Yin H., Kanasty R.L., Eltoukhy A.A., Vegas A.J., Dorkin J.R., Anderson D.G. (2014). Non-viral vectors for gene-based therapy. Nat. Rev. Genet..

[14] Garnett M.C. (1999). Gene-delivery systems using cationic polymers. Crit. Rev. Ther. Drug Carrier Syst..

[15] Kichler A., Leborgne C., Coeytaux E., Danos O. (2001). Polyethylenimine-mediated gene delivery: A mechanistic study. J. Gene Med..

[16] Abdallah B., Hassan A., Benoist C., Goula D., Behr J.P., Demeneix B.A. (1996). A powerful nonviral vector for in vivo gene transfer into the adult mammalian brain: Polyethylenimine. Hum. Gene Ther..

[17] Sweeney P., Karashima T., Ishikura H., Wiehle S., Yamashita M., Benedict W.F., Cristiano R.J., Dinney C.P. (2003). Efficient therapeutic gene delivery after systemic administration of a novel polyethylenimine/DNA vector in an orthotopic bladder cancer model. Cancer Res..

[18] Boussif O., Lezoualc’h F., Zanta M.A., Mergny M.D., Scherman D., Demeneix B., Behr J.P. (1995). A versatile vector for gene and oligonucleotide transfer into cells in culture and in vivo: Polyethylenimine. Proc. Natl. Acad. Sci. USA.

[19] Venkiteswaran S.T.T., Thomas T.J. (2016). Selectivity of polyethyleneimines on DNA nanoparticle preparation and gene transport. ChemistrySelect.

[20] Lungwitz U., Breunig M., Blunk T., Gopferich A. (2005). Polyethylenimine-based non-viral gene delivery systems. Eur. J. Pharm. Biopharm..

[21] Urban-Klein B., Werth S., Abuharbeid S., Czubayko F., Aigner A. (2005). RNAi-mediated gene-targeting through systemic application of polyethylenimine (PEI)-complexed siRNA in vivo. Gene Ther..

[22] Zintchenko A., Philipp A., Dehshahri A., Wagner E. (2008). Simple modifications of branched PEI lead to highly efficient siRNA carriers with low toxicity. Bioconjugate Chem..

[23] Fischer D., Li Y., Ahlemeyer B., Krieglstein J., Kissel T. (2003). In vitro cytotoxicity testing of polycations: Influence of polymer structure on cell viability and hemolysis. Biomaterials.

[24] Godbey W.T., Wu K.K., Mikos A.G. (1999). Size matters: Molecular weight affects the efficiency of poly(ethylenimine) as a gene delivery vehicle. J. Biomed. Mater. Res..

[25] He Y., Cheng G., Xie L., Nie Y., He B., Gu Z. (2013). Polyethyleneimine/DNA polyplexes with reduction-sensitive hyaluronic acid derivatives shielding for targeted gene delivery. Biomaterials.

[26] Kurosaki T., Kitahara T., Fumoto S., Nishida K., Nakamura J., Niidome T., Kodama Y., Nakagawa H., To H., Sasaki H. (2009). Ternary complexes of pDNA, polyethylenimine, and gamma-polyglutamic acid for gene delivery systems. Biomaterials.

[27] Zhou X., Li X., Gou M., Qiu J., Li J., Yu C., Zhang Y., Zhang N., Teng X., Chen Z. (2011). Antitumoral efficacy by systemic delivery of heparin conjugated polyethylenimine-plasmid interleukin-15 complexes in murine models of lung metastasis. Cancer Sci..

[28] Shih I.L., Van Y.T. (2001). The production of poly-(gamma-glutamic acid) from microorganisms and its various applications. Bioresour. Technol..

[29] Peng S.F., Yang M.J., Su C.J., Chen H.L., Lee P.W., Wei M.C., Sung H.W. (2009). Effects of incorporation of poly(gamma-glutamic acid) in chitosan/DNA complex nanoparticles on cellular uptake and transfection efficiency. Biomaterials.

[30] Peng S.F., Tseng M.T., Ho Y.C., Wei M.C., Liao Z.X., Sung H.W. (2011). Mechanisms of cellular uptake and intracellular trafficking with chitosan/DNA/poly(gamma-glutamic acid) complexes as a gene delivery vector. Biomaterials.

[31] Liao Z.X., Peng S.F., Ho Y.C., Mi F.L., Maiti B., Sung H.W. (2012). Mechanistic study of transfection of chitosan/DNA complexes coated by anionic poly(gamma-glutamic acid). Biomaterials.

[32] Liao Z.X., Ho Y.C., Chen H.L., Peng S.F., Hsiao C.W., Sung H.W. (2010). Enhancement of efficiencies of the cellular uptake and gene silencing of chitosan/siRNA complexes via the inclusion of a negatively charged poly(gamma-glutamic acid). Biomaterials.

[33] Wang Y., Gao S., Ye W.H., Yoon H.S., Yang Y.Y. (2006). Co-delivery of drugs and DNA from cationic core-shell nanoparticles self-assembled from a biodegradable copolymer. Nat. Mater..

[34] Qiu L.Y., Bae Y.H. (2007). Self-assembled polyethylenimine-graft-poly(epsilon-caprolactone) micelles as potential dual carriers of genes and anticancer drugs. Biomaterials.

[35] Lu S., Morris V.B., Labhasetwar V. (2015). Codelivery of DNA and siRNA via arginine-rich PEI-based polyplexes. Mol. Pharm..

[36] Bishop C.J., Tzeng S.Y., Green J.J. (2015). Degradable polymer-coated gold nanoparticles for co-delivery of DNA and siRNA. Acta Biomater..

[37] Shim M.S., Kwon Y.J. (2011). Dual mode polyspermine with tunable degradability for plasmid DNA and siRNA delivery. Biomaterials.

[38] Felgner P.L., Gadek T.R., Holm M., Roman R., Chan H.W., Wenz M., Northrop J.P., Ringold G.M., Danielsen M. (1987). Lipofection: A highly efficient, lipid-mediated DNA-transfection procedure. Proc. Natl. Acad. Sci. USA.

[39] Peng S.F., Su C.J., Wei M.C., Chen C.Y., Liao Z.X., Lee P.W., Chen H.L., Sung H.W. (2010). Effects of the nanostructure of dendrimer/DNA complexes on their endocytosis and gene expression. Biomaterials.

[40] Malmo J., Sorgard H., Varum K.M., Strand S.P. (2012). siRNA delivery with chitosan nanoparticles: Molecular properties favoring efficient gene silencing. J. Control. Release.

[41] Thomas T.J., Tajmir-Riahi H.A., Thomas T. (2016). Polyamine-DNA interactions and development of gene delivery vehicles. Amino Acids.

[42] Vijayanathan V., Thomas T., Shirahata A., Thomas T.J. (2001). DNA condensation by polyamines: A laser light scattering study of structural effects. Biochemistry.

[43] Akinc A., Thomas M., Klibanov A.M., Langer R. (2005). Exploring polyethylenimine-mediated DNA transfection and the proton sponge hypothesis. J. Gene Med..

[44] Sonawane N.D., Szoka F.C., Verkman A.S. (2003). Chloride accumulation and swelling in endosomes enhances DNA transfer by polyamine-DNA polyplexes. J. Biol. Chem..

[45] Chen J., Lin L., Guo Z., Xu C., Li Y., Tian H., Tang Z., He C., Chen X. (2016). *N*-Isopropylacrylamide Modified Polyethylenimines as Effective siRNA Carriers for Cancer Therapy. J. Nanosci. Nanotechnol..

[46] Das J., Das S., Paul A., Samadder A., Bhattacharyya S.S., Khuda-Bukhsh A.R. (2014). Assessment of drug delivery and anticancer potentials of nanoparticles-loaded siRNA targeting STAT3 in lung cancer, in vitro and in vivo. Toxicol. Lett..

[47] Schaffert D., Ogris M. (2013). Nucleic acid carrier systems based on polyethylenimine conjugates for the treatment of metastatic tumors. Curr. Med. Chem..

[48] Li S., Rizzo M.A., Bhattacharya S., Huang L. (1998). Characterization of cationic lipid-protamine-DNA (LPD) complexes for intravenous gene delivery. Gene Ther..

[49] Howard K.A. (2009). Delivery of RNA interference therapeutics using polycation-based nanoparticles. Adv. Drug Deliv. Rev..

[50] Grayson A.C., Doody A.M., Putnam D. (2006). Biophysical and structural characterization of polyethylenimine-mediated siRNA delivery in vitro. Pharm. Res..

[51] Zhang S., Zhou L., Hong B., van den Heuvel A.P., Prabhu V.V., Warfel N.A., Kline C.L., Dicker D.T., Kopelovich L., El-Deiry W.S. (2015). Small-Molecule NSC59984 Restores p53 Pathway Signaling and Antitumor Effects against Colorectal Cancer via p73 Activation and Degradation of Mutant p53. Cancer Res..

[52] Zhong Z., Feijen J., Lok M.C., Hennink W.E., Christensen L.V., Yockman J.W., Kim Y.H., Kim S.W. (2005). Low molecular weight linear polyethylenimine-*b*-poly(ethylene glycol)-*b*-polyethylenimine triblock copolymers: Synthesis, characterization, and in vitro gene transfer properties. Biomacromolecules.

[53] Kodama Y., Shiokawa Y., Nakamura T., Kurosaki T., Aki K., Nakagawa H., Muro T., Kitahara T., Higuchi N., Sasaki H. (2014). Novel siRNA delivery system using a ternary polymer complex with strong silencing effect and no cytotoxicity. Biol. Pharm. Bull..

[54] Rejman J., Bragonzi A., Conese M. (2005). Role of clathrin- and caveolae-mediated endocytosis in gene transfer mediated by lipo- and polyplexes. Mol. Ther..

[55] Gabrielson N.P., Pack D.W. (2009). Efficient polyethylenimine-mediated gene delivery proceeds via a caveolar pathway in HeLa cells. J. Control. Release.

[56] Hwang M.E., Keswani R.K., Pack D.W. (2015). Dependence of PEI and PAMAM Gene Delivery on Clathrin- and Caveolin-Dependent Trafficking Pathways. Pharm. Res..

[57] Nguyen J., Szoka F.C. (2012). Nucleic acid delivery: The missing pieces of the puzzle?. Acc. Chem. Res..

[58] Vijayanathan V., Thomas T., Thomas T.J. (2002). DNA nanoparticles and development of DNA delivery vehicles for gene therapy. Biochemistry.

[59] Liu M., Huang G., Cong Y., Tong G., Lin Z., Yin Y., Zhang C. (2015). The preparation and characterization of micelles from poly(gamma-glutamic acid)-graft-poly(l-lactide) and the cellular uptake thereof. J. Mater. Sci. Mater. Med..

[60] Koeda S., Ichiki K., Iwanaga N., Mizuno K., Shibata M., Obata A., Kasuga T., Mizuno T. (2016). Construction and Characterization of Protein-Encapsulated Electrospun Fibermats Prepared from a Silica/Poly(gamma-glutamate) Hybrid. Langmuir.

[61] Maya S., Sarmento B., Lakshmanan V.K., Menon D., Jayakumar R. (2014). Actively targeted cetuximab conjugated gamma-poly(glutamic acid)-docetaxel nanomedicines for epidermal growth factor receptor over expressing colon cancer cells. J. Biomed. Nanotechnol..

[62] Chu Y.L., Ho C.T., Chung J.G., Raghu R., Lo Y.C., Sheen L.Y. (2013). Allicin induces anti-human liver cancer cells through the p53 gene modulating apoptosis and autophagy. J. Agric. Food Chem..

